# Analysis of *Pseudomonas aeruginosa* Isolates from Patients with Cystic Fibrosis Revealed Novel Groups of Filamentous Bacteriophages

**DOI:** 10.3390/v15112215

**Published:** 2023-11-05

**Authors:** Peter Evseev, Julia Bocharova, Dmitriy Shagin, Igor Chebotar

**Affiliations:** 1 Laboratory of Molecular Microbiology, Pirogov Russian National Research Medical University, Ostrovityanova 1, 117997 Moscow, Russia; ivrin7@gmail.com (J.B.); shagdim777@gmail.com (D.S.); 2 Laboratory of Molecular Bioengineering, Shemyakin-Ovchinnikov Institute of Bioorganic Chemistry, Russian Academy of Sciences, Miklukho-Maklaya 16/10, 117997 Moscow, Russia

**Keywords:** filamentous bacteriophages, Pf, Pseudomonas phages, *Pseudomonas aeruginosa*, bacteriophage taxonomy

## Abstract

*Pseudomonas aeruginosa* is an opportunistic pathogen that can cause infections in humans, especially in hospital patients with compromised host defence mechanisms, including patients with cystic fibrosis. Filamentous bacteriophages represent a group of single-stranded DNA viruses infecting different bacteria, including *P. aeruginosa* and other human and animal pathogens; many of them can replicate when integrated into the bacterial chromosome. Filamentous bacteriophages can contribute to the virulence of *P. aeruginosa* and influence the course of the disease. There are just a few isolated and officially classified filamentous bacteriophages infecting *P. aeruginosa*, but genomic studies indicated the frequent occurrence of integrated prophages in many *P. aeruginosa* genomes. An analysis of sequenced genomes of *P. aeruginosa* isolated from upper respiratory tract (throat and nasal swabs) and sputum specimens collected from Russian patients with cystic fibrosis indicated a higher diversity of filamentous bacteriophages than first thought. A detailed analysis of predicted bacterial proteins revealed prophage regions representing the filamentous phages known to be quite distantly related to known phages. Genomic comparisons and phylogenetic studies enabled the proposal of several new taxonomic groups of filamentous bacteriophages.

## 1. Introduction

Cystic fibrosis (CF) is a genetic disease that develops as a result of mutations in the cystic fibrosis transmembrane conductance regulator protein (CFTR) gene [1]. Breakdowns in CFTR lead to the disruption of the transport of chlorine ions through the membrane, resulting in significant pathological changes at the level of the mucous membranes of the lungs, pancreas, intestines, and kidneys [2]. This is accompanied by the suppression of local immunity and the expansion of opportunistic flora and global metabolic disorders, which ultimately leads to a catastrophic reduction in the life of patients. The most common manifestation of CF is pulmonary disease. The key driver of pulmonary disease is a chronic infectious process, one of the main pathogens of which is *Pseudomonas aeruginosa* [3]. The *P. aeruginosa* arsenal includes many virulence factors including products that increase the viscosity of bronchial epithelial secretions, which aggravates impaired bronchial drainage and increases inflammation. These are alginate components of the biofilm matrix (Psl, Pel), as well as products of filamentous bacteriophages infecting *P. aeruginosa* [4,5].

Filamentous bacteriophages are a group of single-stranded DNA (ssDNA) bacterial viruses (bacteriophages, phages) possessing unique morphological and genomic features. Virions of filamentous bacteriophages are worm-shaped, long, flexible hollow tubes composed of several thousand major capsid protein subunits containing genomic ssDNA [6,7]. Filamentous phages can infect both Gram-negative and Gram-positive bacteria; they can replicate by being integrated into the chromosome of a bacterial host or adhere to non-integrative episomal replication [6,7,8]. These viruses constitute the order *Tubulavirales*, containing temperate, morphologically similar phages assigned to three families that include 31 genera (as of July 2023). The phages infecting *Pseudomonas aeruginosa* (Pf phages) are assigned to the *Inoviridae* family.

The analysis of bacterial genomes indicated the prevalence of filamentous (pro)phages infecting *P. aeruginosa* (Pf (pro)phages) among different *P. aeruginosa* isolates [9]. They can be found within the genomic sequences of *P. aeruginosa* contained in open databases: more than half of the genomes in the Pseudomonas Genome Database (www.pseudomonas.com, accessed on 15 August 2023) contain evidence of the presence of integrated Pf sequences [10]. The most common prophages were found to be Pf1-like genetic elements (including Pf1, Pf4, Pf5) [9], and they are widely used as model viruses in research studies [8]. So far, the International Committee on Taxonomy of Viruses (ICTV) has recognised three *P. aeruginosa* Pf phages: *Primolicivirus Pf1*, *Primolicivirus Pf8,* and *Tertilicivirus Pf3* (https://ictv.global/taxonomy, accessed on 15 August 2023), although the diversity of Pf phages is higher; eight phages (Pf1–Pf7 and Pf-LESB5) have been identified, although phage Pf2 is no longer mentioned in the literature [8,11,12]. Pf1-like phages Pf1, Pf4-Pf7, and Pf-LESB5 demonstrate distinct genetic similarity, while the phage Pf3 is distant from them [6,7].

The bioinformatic methods used in a recent meticulous study carried out in search of Pf genetic elements in *P. aeruginosa* genomes were based on the homology searches using BLAST v2.13.0+ tools and known Pf sequences [13]. However, the content in genetic databases is becoming increasingly diverse, and viral genomic research studies employing more advanced remote homology search methods allow the detection of new viruses and viral groups often distantly related to known viruses and groups [14,15]. Here, a thorough bioinformatic analysis was performed using the genomic data obtained using sputum samples from patients with cystic fibrosis. The purpose of the present study was to reveal and characterise the filamentous prophages in the genomes of *P. aeruginosa* contained in the samples in order to propose the taxonomy and phylogeny of corresponding phages and reveal their evolutionary relations with known groups of phages.

## 2. Materials and Methods

### 2.1. Sample Collection, Bacterial Genome Sequencing and Annotation

*P. aeruginosa* isolates were recovered from upper respiratory tract (throat and nasal swabs) and sputum specimens collected from Russian patients with cystic fibrosis in January–February 2020. Samples were inoculated to agar plates no later than one hour after sample collection. Sample processing and isolation of microorganisms were performed according to Laboratory standards for processing microbiological samples from people with cystic fibrosis (https://www.cysticfibrosis.org.uk/sites/default/files/2020-12/Laboratory%20standards.pdf, accessed on 15 August 2023) using UriSelect4 Agar and Pseudomonas aeruginosa Agar (Bio-Rad Laboratories, Hercules, CA, USA). MALDI-TOF mass-spectrometry (Vitek-MS, bioMereux, Marcy-l’Etoile, France) was used for species identification. P. aeruginosa isolates were collected and stored at −80 °C. More detailed characterisation of *P. aeruginosa* isolates is presented in a previous study [16].

In total, 88 *P. aeruginosa* clinical isolates were whole genome sequenced (WGS). Bacterial DNA was isolated from an overnight culture using a QIAamp DNA Mini Kit (Qiagen, Düsseldorf, Germany). DNA libraries were prepared using an MGIEasy Universal DNA Library Prep Set (MGI Tech, Shenzhen, China) according to the manufacturer’s instructions. DNA (300–600 ng) was fragmented using a Covaris S220 ultrasonicator (Covaris, Woburn, MA, USA). The average fragment length was 250 b.p. DNA libraries were washed with Agencourt AMPure XP (Beckman Coulter, Brea, CA, USA). Concentrations of DNA and DNA libraries were measured with a Qubit Flex instrument (Thermo Fisher Scientific, Waltham, MA, USA) using the dsDNA HS Assay Kit according to the manufacturer’s instructions. The DNA library quality control (determination of the size distribution of the final library and confirmation of the absence of the remaining adapter-dimers) was performed using a Bioanalyzer 2100 and a High Sensitivity DNA kit (Agilent Technologies, Santa Clara, CA, USA). After the circularization procedure, DNA libraries were sequenced on the DNBSEQ-G400 platform in a 100 b.p. pair-end mode with the use of a DNBSEQ-G400RS High-throughput Sequencing Set PE100 kit (MGI Tech) according to the manufacturer instructions. Basecall Lite software G400 (MGI Tech) was used for the FASTQ file generation.

De novo genome assembly was performed using CLC Genomic Workbench 23 (QIAGEN, Aarhus, Denmark) with default settings. Binning was carried out using MetaBAT v2.15 [17]. Assessment of completeness and contamination scores were performed using CheckM v1.0.13 [18]. Bacterial genomes were annotated using Prokka v1.14.5 [19] with default settings.

### 2.2. Search for Integrated Filamentous Prophages

The BLAST v2.13.0+ search (E-value < 10^−3^) [20] for prophage regions representing filamentous phages was performed using amino acid sequences of major capsid protein (MCP) of known sequenced filamentous phages deposited in the NCBI GenBank PHG database (https://www.ncbi.nlm.nih.gov/genbank, accessed on 15 August 2023) and custom BLAST database was constructed using BLAST tools. The MCP sequences used for the search were extracted using phage genome annotations or found manually using Glimmer v3.0.2 [21] for gene predictions, BLAST (E-value < 10^−3^) and HHpred (probability > 90%) [22] for checking the gene function. The BLAST search employed the NCBI nr/nt databases, and the HHpred search used PDB70_mmcif_2023-06-18, PfamA-v35, UniProt-SwissProt-viral70_3_Nov_2021 and NCBI_Conserved_Domains (CD)_v3.19 databases. The remote homology search was accomplished using HH-suite v3.3.0 [23] and query sequences of proteins of 35–110 aa length, predicted in the obtained *P. aeruginosa* genomes. The HH-suite search was performed using hhblits and PDB70_mmcif_2023-06-18 and PfamA-v35 databases applying default settings. Then, the corresponding genomic regions were checked for the presence of adjacent genes encoding other proteins characteristic of the filamentous phage.

### 2.3. Prophage Genomic Analysis

The boundaries of prophage regions were determined by comparing loci containing prophage elements with homologous loci in bacterial chromosomes that do not contain integrated phage genomes found using BLAST. Protein-enclosing genes were predicted with Prokka v1.14.5, Glimmer, Geneious Prime v2023.2.1 (Biomatters, Inc., Auckland, New Zealand) and curated manually taking into account functions of proteins, encoded by possible open-reading frames (ORFs). Functions of putative proteins were predicted by the sequence search using BLAST and the remote homology search using HHpred, as described in Section 2.2. Intergenomic comparisons and calculations of intergenomic similarities were performed using clinker [24] and VIRIDIC [25] with default settings. Genetic maps and gene comparisons were visualised in clinker. Protein sequence alignments were made using Clustal Omega [26] and “number of refinement iterations 3, evaluate full distance matrix for initial guide tree, evaluate full distance matrix for refinement iteration guide tree” command line parameters. Phylogenetic trees were constructed using IQ-TREE v2.2.5 [27] and “--alrt 1000 -B 5000” command line parameters. The resulting consensus trees with bootstrap support values (1000 replicas) were visualised using the iTOL v6 [28].

## 3. Results

### 3.1. Collection of Genomic Data of Known Pf Phages

According to official ICTV nomenclature, filamentous phages are classified as members of the realm *Monodnaviria*, kingdom *Loebvirae*, phylum *Hofneiviricota*, class *Faserviricetes,* and order *Tubulavirales*. As of July 2023, the NCBI phage GenBank database contained 155 nucleotide sequences of filamentous phages, including 139 complete genomes. Of these, 131 complete genomes, including phages infecting *Pseudomonas* and other Gammaproteobacteria, were classified as members of the *Inoviridae* family, two *Thermus* phages were classified as members of the *Paulinoviridae* family, and seven phages infecting cell wall-less bacteria were assigned to the *Plectroviridae* family. The *Pseudomonas* phage sequences recognised by the ICTV comprised genomic sequences of phages Pf1 (*Primolicivirus Pf1*), Pf3 (*Tertilicivirus Pf3*), and Pf8 (*Primolicivirus Pf8*). Sequences of *Pseudomonas* phages Pf4-Pf7 and Pf-LESB5 were extracted from host bacterial sequences, as well as prophage sequences pf Pf1-like phages Pf Nhmuc and Pf paerg010 analysed in a recent study [8]. Since the genomic bacterial sequence of *P. aeruginosa* isolate paerg010 was found to contain two Pf prophage regions, the second prophage, in addition to the one analysed in [8] and labelled here as Pf paerg010-2, was added to the list of Pf phages used for comparisons in the present study (Table 1). The boundaries of the prophage regions were established according to [8] and using GenBank annotations and the locations of terminal repeats.

### 3.2. General Characterisation of Pf prophages Found in Genomic Sequences of P. aeruginosa Present in Clinical Isolates

To reveal the Pf phages both related to and distant from the described Pf phages, both BLAST homology and HH-suite remote homology searches were used. The combined search revealed a total of 73 Pf prophage regions in 56 out of 88 isolates’ genomes (Supplementary Table S1) that contained genes encoding proteins characteristic of filamentous phages, including coat proteins (major capsid protein CoaB and minor capsid protein, or attachment protein CoaA), the replication initiation protein (RP), and the assembly protein (“ZOT-like”), which show a distant similarity to *Vibrio cholerae* zonula occludens toxin (ZOT) [29]. Clustering of the identified prophages using the CoaB amino acid sequences united them into seven groups (Supplementary Figure S1), and the genomic organisation within each group was similar (as well as between some groups). Seven representative sequences were taken for further analysis (Table 2, Supplementary File S1). Next, we will refer to these representative (pro)phages by the name of the isolates in whose genomes they were found: Group 1—Pf 54-4, Group 2—Pf 99-2, Group 3—Pf 62-2, Group 4—Pf 36-2, Group 5—Pf 42-1, Group 6—Pf 97-1, and Group 7—Pf 17897. These sequences were deposited in the NCBI GenBank under Accession numbers OR621019–OR621025.

What all representative prophages have in common is that their GC content (54.7–59.5%) is lower than that of the bacterial genomes (66.1–66.6%) in which they were found. The difference in GC content is higher than 10% for Pf 54-4, Pf 99-2, Pf 62-2, and Pf 17897. A comparison with previously described Pf phages (Table 1) indicates that this may be a common feature for all Pf phages. Interestingly, phage Pf3, which is genetically completely different from the other Pf phages, exhibits the lowest GC content of 45.4%, while the well-characterised phage Pf1 has the highest GC of 61.5%. Together with the frequent occurrence of Pf1-like phages, this may indicate that these phages have a longer history of host–parasite relations than the phage Pf3. The size of the prophage regions of Pf 54-4, Pf 99-2, and Pf 62-2, representing groups 1–3, is nearly twice as large as that of the remaining phages (12,602–13,538 bp vs. 6654–7418 bp), and the number of predicted ORFs in these prophages is also higher (15–18 vs. 9–12).

**Figure 1:**
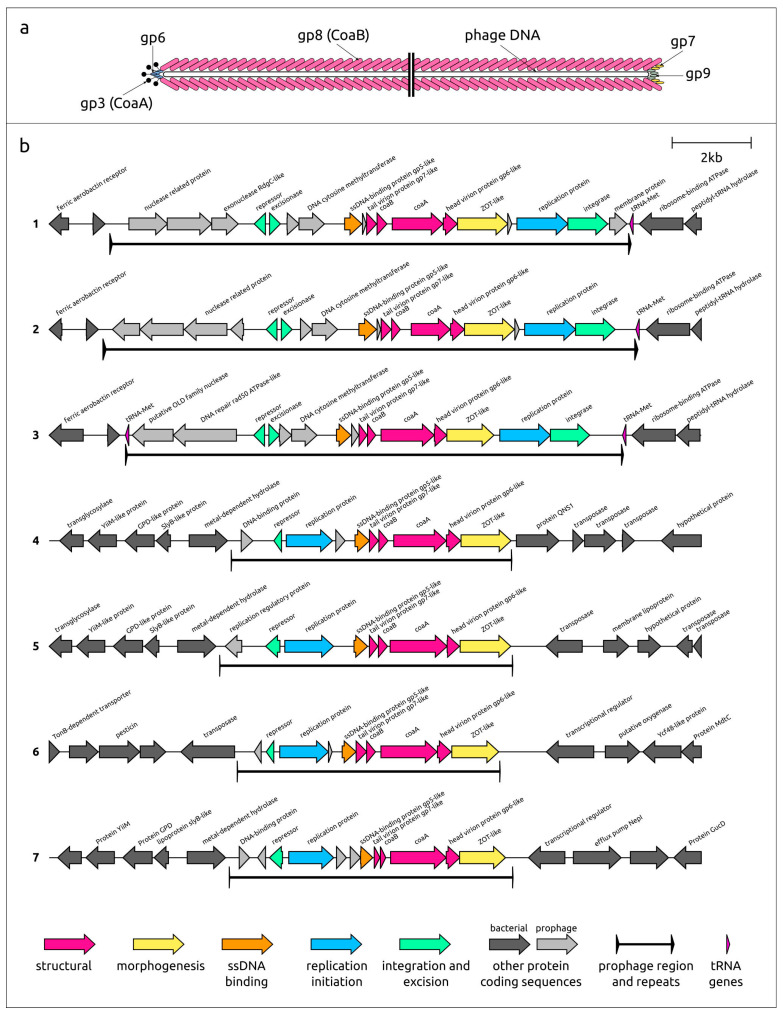
(**a**) Schematic view of a filamentous phage showing the overall architecture of virion according to [6,32,34]; (**b**) Genetic maps of seven representative prophages predicted to be contained in analysed genomes of *P. aeruginosa*. The number to the left of genetic scheme indicates the representative prophage region: 1—the Pf 54-4; 2—Pf 99-2; 3—Pf 62-2; 4—Pf 36-2, 5—Pf 42-1, 6—Pf 97-1, 7—Pf 17897. Arrows indicate the direction of transcription. Gene functions and boundaries of prophage regions are shown in labels and legends.

The genomic organisation within the prophage regions of Pf 54-4, Pf 99-2, and Pf 62-2 is similar (Figure 1). It seems that the genomes of corresponding phages encode an integrase, an excisionase, and a repressor involved in the regulation of lysogeny. In prophage regions, adjacent back-to-back excisionase and repressor genes are located upstream of structural and morphogenesis genes, while the integrase gene is positioned downstream at the end (3′-part of the leading strain) of the prophage region.

The architecture of morphogenesis and structural gene blocks is similar in all seven representative prophages. Like in other filamentous phages, this block includes a single-stranded (ss) DNA-binding protein, presumably covering phage ssDNA during the assembly of virions, replaced by major capsid protein CoaB [30]. The block also includes minor capsid protein or attachment protein CoaA, which acts as a receptor-binding protein necessary for the pilus-mediated adsorption of the phage into the bacterial cell [31]. The list of genes encoding structural proteins includes a gene encoding the tail protein, located between the genes of ssDNA-binding and capsid proteins, and the head virion protein gene, located downstream of the *coaA* gene. Interestingly, no genes encoding analogues of tail protein gp9 (also called p9 or pIX, Figure 1a), present in some known filamentous phages [6,32], have been found. This may be related to the high level of divergence of this protein during evolution; it can be assumed that the ORF that may encode this gene is located near another gene encoding the gp7-like virion tail protein (applying the notation used in [6,32]). In all representative prophage regions, the morphogenesis and structural genes block ends with the ZOT-like virion assembly protein gene. An HHpred search revealed a clear similarity between the N-terminal part of the predicted ZOT-like proteins and the ZOT protein encoded in the genome of *Vibrio* phage CTXφ. The N-terminal region of CTXφ ZOT protein is involved in the morphogenesis of the phage, while the C-terminal part may be responsible for the ZOT enterotoxic activity [29,33].

The genetic architecture of the newly identified Pf prophage regions is quite different for Pf 36-2, Pf 42-1, Pf 97-1, and Pf 17897, representing groups 4–7. (Table 2). This architecture has not previously been described for Pf prophage regions and is characterised by the absence of integrase and excisionase genes. However, these prophage regions contain a gene encoding the proteins similar to phage, transposon, and plasmid repressors, and that is possibly involved in the lysogeny regulation (Figure 1). Thus, new phages can be divided into two large groups, which may differ in the mechanism of integration into the host genome.

### 3.3. Intergenomic Comparisons and Phylogenetic Analysis

#### 3.3.1. Genome Alignment

**Figure 2:**
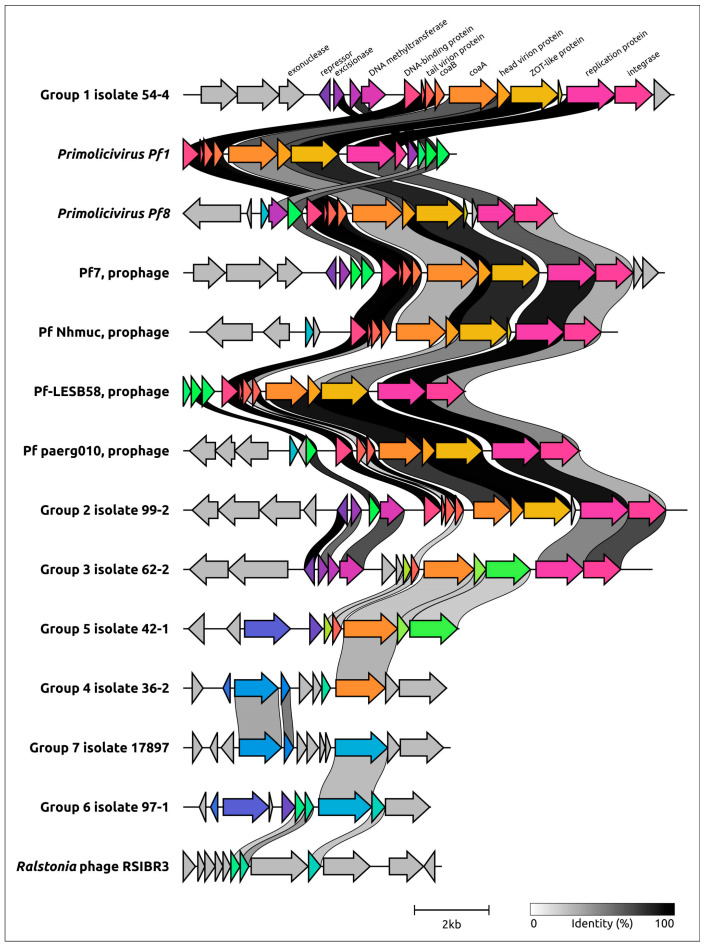
Comparative genome alignment of filamentous prophages newly identified in analysed genomes of *P. aeruginosa* and known (pro)phages *Pseudomonas* Pf1 (*Primolicivirus Pf1*), Pf8 (*Primolicivirus Pf8*), Pf7, Pf Nhmuc, Pf-LESB58, Pf paerg010, *Ralstonia* phage RSIBR3. Percent amino acid identity is represented as greyscale links between genomes. Homologous proteins are assigned a unique colour. Arrows indicate the direction of transcription.

The comparative genome alignment of previously described Pf and newly identified prophage regions containing Pf genetic elements indicates similarity in the genetic organisation and proteins of phages Pf 54-4, Pf 99-2, Pf 62-2 (groups 1–3), and Pf1-like phages (Figure 2). This primarily concerns genes encoding structural, morphogenetic, and replication proteins. The level of conservation of protein sequences varies. The minor capsid protein CoaA appears to be less similar between different Pf1-like phages and phages Pf 54-4, Pf 99-2, and Pf 62-2. This may be due to the lesser similarity of receptor-binding proteins that are evolutionarily adapted to different hosts. Also, it appears that the genomes of Pf phages are characterised by pronounced mosaicism when some genes and even genomic modules of related phages are dissimilar due to the horizontal transfer of their functional analogues [35,36,37].

#### 3.3.2. VIRIDIC Analysis

**Figure 3:**
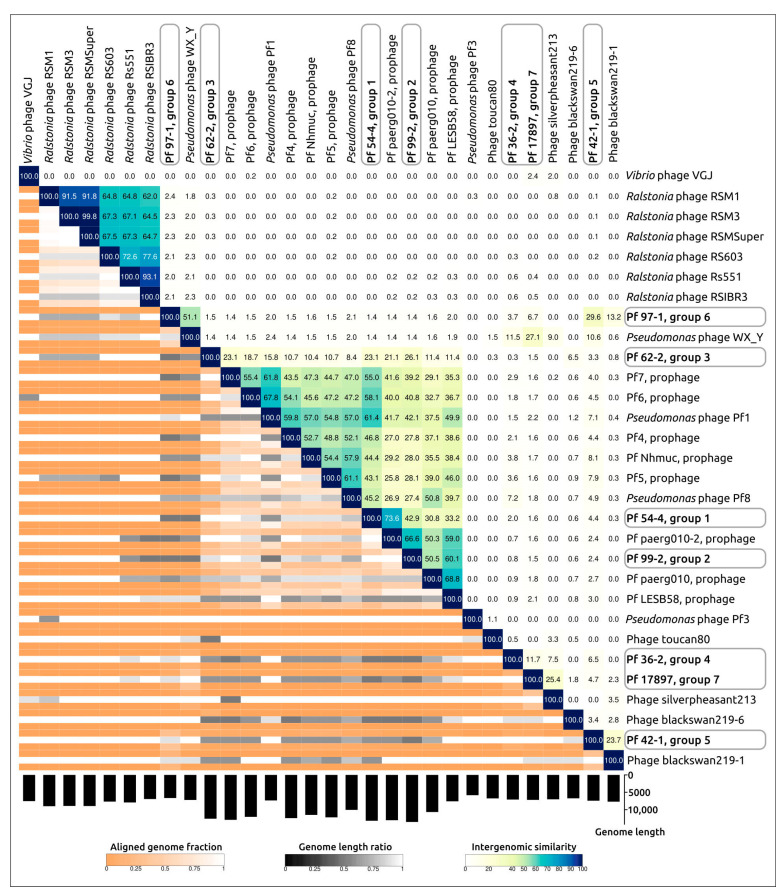
VIRIDIC-generated heatmap of seven representative prophages and related filamentous phages. The colour coding in the upper right part of the map indicates the clustering of the phage genomes based on intergenomic similarity. Numbers represent similarity values for each genome pair, rounded to the first decimal. The aligned genome fraction and genome length ratio are shown in the lower left of the map using a colour gradient in the legends. The length of prophage regions is shown in the histogram below the map.

The intergenomic comparison was performed using the VIRIDIC tool used by ICTV as a primary classification technique. The VIRIDIC analysis was conducted using all *Tubulavirales* genomic sequences contained in the Phage GenBank database, including Pf phages and excluding duplicates and partial sequences (Supplementary Figure S2), as well as Pf phages and several other phages distantly related to new groups (Figure 3). Additionally, a BLAST search using the sequences of the structural proteins revealed an apparently misclassified *Pseudomonas* phage WX_Y, attributed to a *Caudoviricetes* virus. The latter phage was also added to samples for VIRIDIC analysis.

The VIRIDIC calculations placed the sequences of Pf 54-4, Pf 99-2, and Pf 62-2 (groups 1–3) into a large cluster containing all known Pf phages except Pf3, but the intergenomic similarity values compared to known Pf phages were lower than the 70% genus threshold [38] for all three prophages. Prophages Pf 36-2 (group 4) and Pf 17897 (group 7) showed some level of similarity (7.5–25.4%) to Phage silverpheasant213, while prophages Pf 42-1 (group 5) and Pf 97-1 (group 6) showed a low level of similarity (13.2–23.7%) to Phage blackswan219-6. Phages blackswan219-6 and silverpheasant213 were detected in the cloacal swab samples of birds using the metagenomics technique [39]. It is possible that these phages are filamentous bacteriophages that infect *Pseudomonas*. Interestingly, VIRIDIC clustering clearly showed the similarity of some Pf sequences to different clusters, which may reflect the genetic mosaicism inherent in many phages, especially temperate ones.

#### 3.3.3. Phylogenetic Analysis

**Figure 4:**
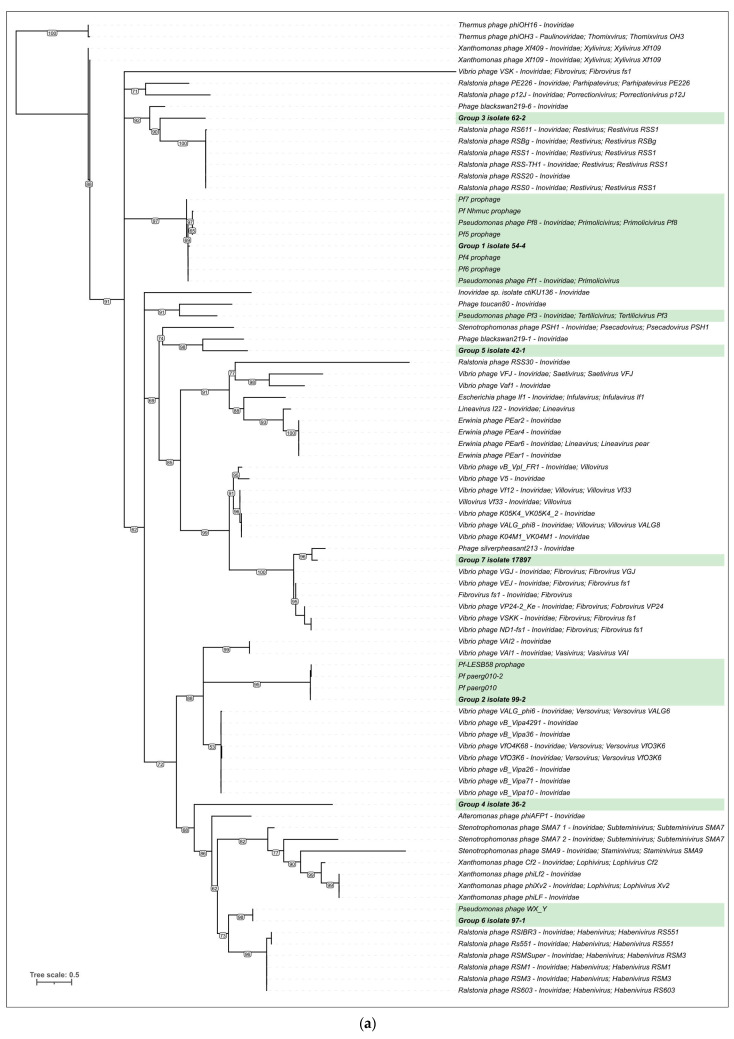

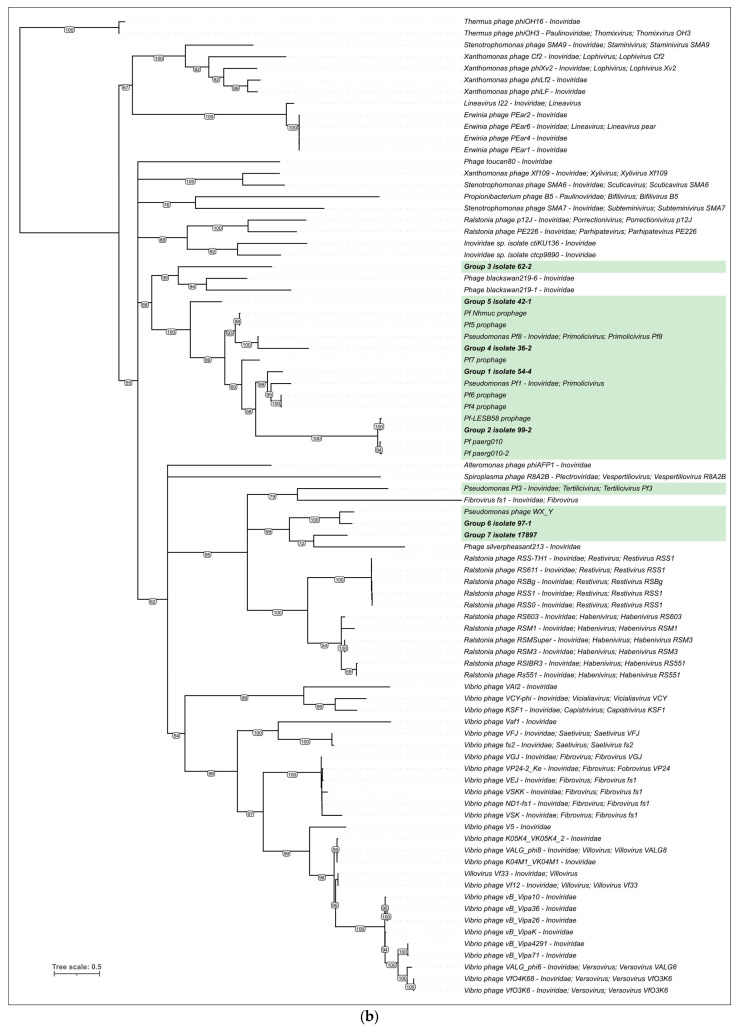

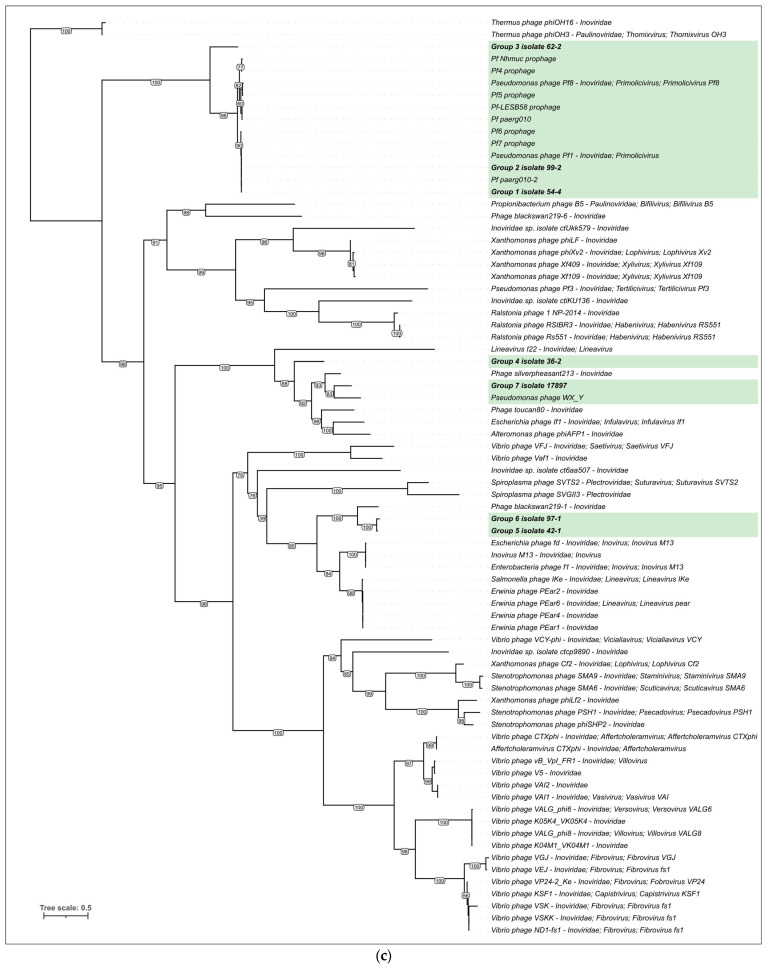

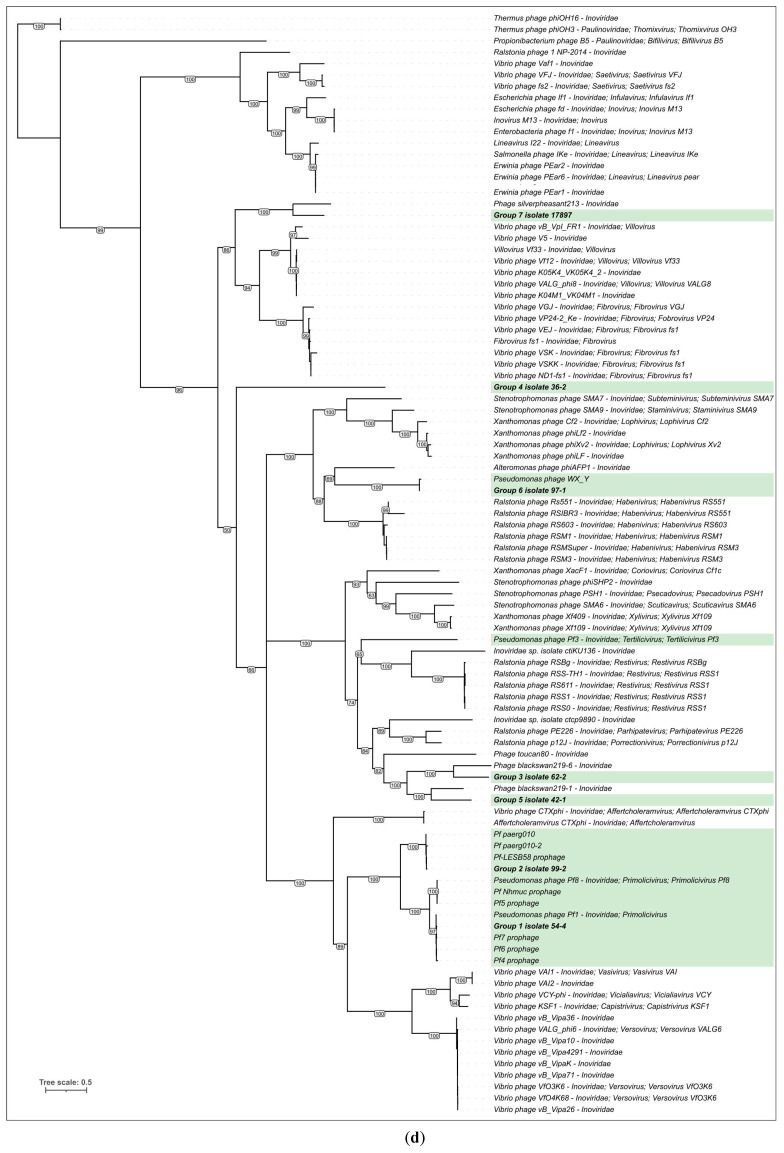
Phylogenetic trees based on amino acid sequences of major capsid protein CoaB (**a**), minor capsid protein CoaA (**b**), replication protein RP (**c**), assembly ZOT-like protein (**d**). Pf phages are highlighted with a green background. Bootstrap values are shown near their branches. Branches with the bootstrap support lower than 50% were deleted. The scale bar shows 0.5 estimated substitutions per site, and the trees were rooted to *Thermus* phages.

The phylogenetic analysis was performed using protein sequences of the major and minor capsid proteins (CoaB and CoaA), the replication protein RP, and the assembly ZOT-like protein (Figure 4). To construct the trees, homologous proteins detected using BLAST and translated sequences of annotated genes contained in the genomes of filamentous phages were used. The topology of the trees is different, which indicates a rich evolutionary history of the formation of the genomes of filamentous phages. The CoaB phylogeny placed only two new Pf prophages together with known Pf phages. Pf 54-4 (group 1) was clustered together with most of the known Pf phages (Pf1, Pf4-Pf8, and Pf Nhmuc), and Pf 99-2 (group 2) was placed in a clade together with the Pf phages integrated in the genomes of *P. aeruginosa* LESB58 and paerg010. Additionally, the CoaB phylogeny placed prophage Pf 97-1 (group 6) and misclassified phage *Pseudomonas* WX_Y in a clade with *Ralstonia* phages classified in the genus *Habenivirus* (family *Inoviridae*). Interestingly, prophage Pf 62-2, representing group 3 of the new Pf phages, was also grouped together with phages infecting *Ralstonia* but belonging to a different *Inoviridae* genus, *Restivirus*. It is possible that the analysed major capsid proteins of the filamentous phages belong to different lineages that infect relatively distant evolutionary bacteria. Other trees also placed new phages in different clades. The CoaA tree collected more Pf phages into one clade, including new ones; this may be due to the receptor-binding function of CoaA. The CoaA, RP, and ZOT trees placed Pf 54-4 and Pf 99-2 in the same branch with Pf1-like phages, indicating the overall closeness between Pf1-like phages and the new prophages of groups 1 and 2.

## 4. Discussion

Filamentous phages are known to increase the virulence of *P. aeruginosa* [10,40]. In particular, the sputum of CF patients induces the self-assembly of biopolymers of filamentous phages into a highly ordered liquid crystal, which makes *P. aeruginosa* biofilms denser and more protected from negative environmental factors [40,41]. The effect of new phages discovered in this work on the virulence of *P. aeruginosa* requires further study.

The results of this study showed a higher diversity of filamentous bacteriophages infecting *P. aeruginosa* than initially thought. The analysis of the genomes of *P. aeruginosa* isolates indicated that most of them (56 out of 88, or 64%) contain integrated filamentous prophage sequences; this result is consistent with previously published data [9,42]. Interestingly, 17 genomes (19%) contain two or more integrated Pf phages. Previous data have shown that known Pf phages (with the exception of Pf3) are similar to the phage Pf1, but the analysis performed in this present study identified both Pf1-like phages and more distantly related groups.

The majority of the Pf prophages detected In the genomes of *P. aeruginosa* during the study (groups 1–3) are similar in their genomic organisation and content to known Pf1-like prophages. They exhibit a detectable level of gene synteny with Pf1-like phages and appear to use phage-encoded integrase and tRNA integration sites like most described Pf1-like phages [5,43]. At the same time, the prophages of groups 4–7 have a different type of genomic organisation. They can be distinguished from Pf1-like prophages by their gene order (with the exception of a structural block common to all filamentous phages), and they do not show homology with most of the genes of Pf1-like (pro)phages and prophages of groups 1–3. The prophage regions assigned to groups 4–6 do not contain the integrase gene; the integration sites of these phages are situated in intergenic regions and do not have homology with tRNA sequences. It may be the case that the phages of groups 4–6 integrate into the bacterial chromosome using the host-encoded integrases.

Following the ICTV approaches, both known and newly found prophages can be classified, applying the 70% genus threshold of nucleotide identity and monophyleticity according to the results of the phylogenetic analysis. The results of intergenomic similarity calculations indicate a significant diversity of Pf phages, which allows many of them to be classified into different taxa according to formal criteria. In particular, the VIRIDIC intergenomic similarity of prophage Pf 62-2 (group 3), similar to the known Pf1-like (pro)phages and new prophages Pf 54-4 (group 1) and Pf 99-2 (group 2) in terms of genome architecture, is only 26.1% and is lower compared to these (pro)phages, which indicates the possibility of assigning Pf 62-2 to a new genus. However, prophages Pf 54-4 and Pf 99-2 demonstrate a higher level of nucleotide identity than known prophages. In turn, prophages Pf 36-2, Pf 42-1, Pf 97-1, and Pf 17897 (groups 4–7) do not show a meaningful intergenomic similarity with known Pf phages.

The taxonomic classification of filamentous phages may be hampered by genetic mosaicism featuring the analysed Pf (pro)phage sequences. Genetic mosaicism is characteristic of at least some viral groups, including eukaryotic viruses and temperate phages [44,45,46,47]. In some cases, the mosaic nature of phage genomes can be traced using protein-wide phylogenetic analysis, indicating the results of modular evolution and the exchange of entire gene blocks [37]. The implications of analysing patterns of modular evolution raise the very practical question of how to classify viruses with a pronounced mosaic genome. In the case of filamentous phages, with genomes that contain about a dozen genes, even a single gene acquired via horizontal transfer can have a significant impact on the overall similarity, including the calculations of nucleotide identity and phylogeny. When classifying filamentous phages, we would suggest that it is reasonable to pay attention first to genome organisation, then to the results of intergenomic comparisons, and then to observations from phylogenetic analysis. We suggest lowering the 70% nucleotide identity threshold for genera to 30–50% and considering phylogenies based on individual core protein sequences rather than a single tree constructed using concatenated alignments.

Based on the results of the genomic analysis and comparisons, as well as complex phylogenetic analysis, we propose classifying phages Pf 54-4 (group 1) and Pf 99-2 (group 2) as representatives of the *Primolicivirus* genus of the *Inoviridae* family, and phages Pf 62-2, Pf 36-2, Pf 42-1, Pf 97-1, and Pf 17897 in new genera. Taking into account the differences in genome organisation, the known Pf1-like phages, phages Pf 54-4, Pf 99-2, and Pf 62-2, can be assigned to a new separate subfamily, and phages Pf 36-2, Pf 42-2, Pf 97-1, and Pf 17897, together with closely related phages, may constitute another subfamily of filamentous phages that infect *P. aeruginosa*. The misclassified *Pseudomonas* phage WX_Y could be assigned to the same genus as Pf 97-1.

## 5. Conclusions

The analysis of *Pseudomonas aeruginosa* isolates from patients with cystic fibrosis using a thorough bioinformatic search for genes encoding the major capsid protein revealed prophage regions corresponding to several new groups of filamentous phages. The results of the intergenomic comparisons and phylogenetic analyses showed that some new prophages different from the known filamentous phages and corresponding phages can be assigned to new taxonomic groups. However, significant genetic mosaicism may preclude consistent classification, requiring the development of thoughtful and valid classification approaches.

## Figures and Tables

**Table 1:** General features of known Pf phages downloaded from the NCBI phage GenBank database and extracted from bacterial genomes downloaded from NCBI GenBank.

Phage Name	Accession	Phage ICTV Classification or Bacterial Strain for Prophages	Genome (Prophage Region) Length, Nucleotides	GC-Content
Pf1	NC_001331.1	*Primolicivirus Pf1*	7349	61.5%
Pf3	M11912.1	*Tertilicivirus Pf3*	5833	45.4%
Pf8	MN710383.1	*Primolicivirus Pf8*	10,061	58.1%
Pf4 prophage	AE004091.2	*P. aeruginosa* PAO1	12,437	56.4%
Pf5 prophage	CP000438.1	*P. aeruginosa* UCBPP-PA14	12,209	58.3%
Pf6 prophage	GQ141978.1	*P. aeruginosa* PAO1 substrain MPAO1	12,066	55.9%
Pf7 prophage	CP000744.1	*P. aeruginosa* PA7	12,933	56.8%
Pf-LESB58 prophage	FM209186.1	*P. aeruginosa* LESB58	7599	60.5%
Pf Nhmuc prophage	CP013479.1	*P. aeruginosa* NHmuc	11,507	58.2%
Pf paerg010	NZ_LR130536.1	*P. aeruginosa* paerg010	10,682	56.3%
Pf paerg010-2	NZ_LR130536.1	*P. aeruginosa* paerg010	13,041	54.9%

**Table 2:** Results of search for filamentous phages in the genomes of clinical isolates of *P. aeruginosa* and general features of identified (pro)phages.

	Group 1	Group 2	Group 3	Group 4	Group 5	Group 6	Group 7
Number of found prophages regions	34	28	2	3	4	1	1
Isolate containing the representative prophage	54-4	99-2	62-2	36-2	42-1	97-1	17,897
Representative prophage region size, nucleotides	13,193	13,538	12,602	7101	7418	6654	7182
Representative prophage GC-content	55.8%	54.7%	55.6%	58.8%	58.9%	59.5%	55.6%
Bacterial host genome GC-content	66.1%	66.4%	66.5%	66.5%	66.4%	66.4%	66.5%
Number of predicted ORFs in representative prophage	18	18	15	10	9	10	12
Size of terminal direct repeats in representative prophage, nucleotides	66	82	51	12	12	11	12
Presence of integrase in representative prophage	Yes	Yes	Yes	No	No	No	No
Integrations site in representative prophage	tRNA	tRNA	tRNA	intergenic region	intergenic region	intergenic region	intergenic region

## Data Availability

Data is contained within the article or Supplementary Material.

## Supplementary Materials

The following supporting information can be downloaded at: https://www.mdpi.com/article/10.3390/v15112215/s1, Figure S1: Phylogenetic trees based on amino acid sequences of major capsid protein CoaB found in prophage regions of *P. aeruginosa* isolates. Different groups of Pf prophages are highlighted with different colours. Bootstrap values are shown near their branches. Branches with the bootstrap support lower than 50% were deleted. The scale bar shows 0.5 estimated substitutions per site, and the trees were rooted to *Thermus* phages; Figure S2: VIRIDIC-generated heatmap of seven representative prophages and *Tubulavirales* phages. The colour coding in the upper right part of the map indicates the clustering of the phage genomes based on intergenomic similarity. Numbers represent similarity values for each genome pair, rounded to the first decimal. The aligned genome fraction and genome length ratio are shown in the lower left of the map using a colour gradient in the legends. The length of prophage regions is shown in the histogram below the map. File S1: Annotated GenBank sequences of new prophages.
