# Supplementary figures and images for: Analysis of Pseudomonas aeruginosa Isolates from Patients with Cystic Fibrosis Revealed Novel Groups of Filamentous Bacteriophages
Source: Viruses. 2023 Nov 5;15(11):2215. doi: 10.3390/v15112215 (PMC10675462; doi:10.3390/v15112215)

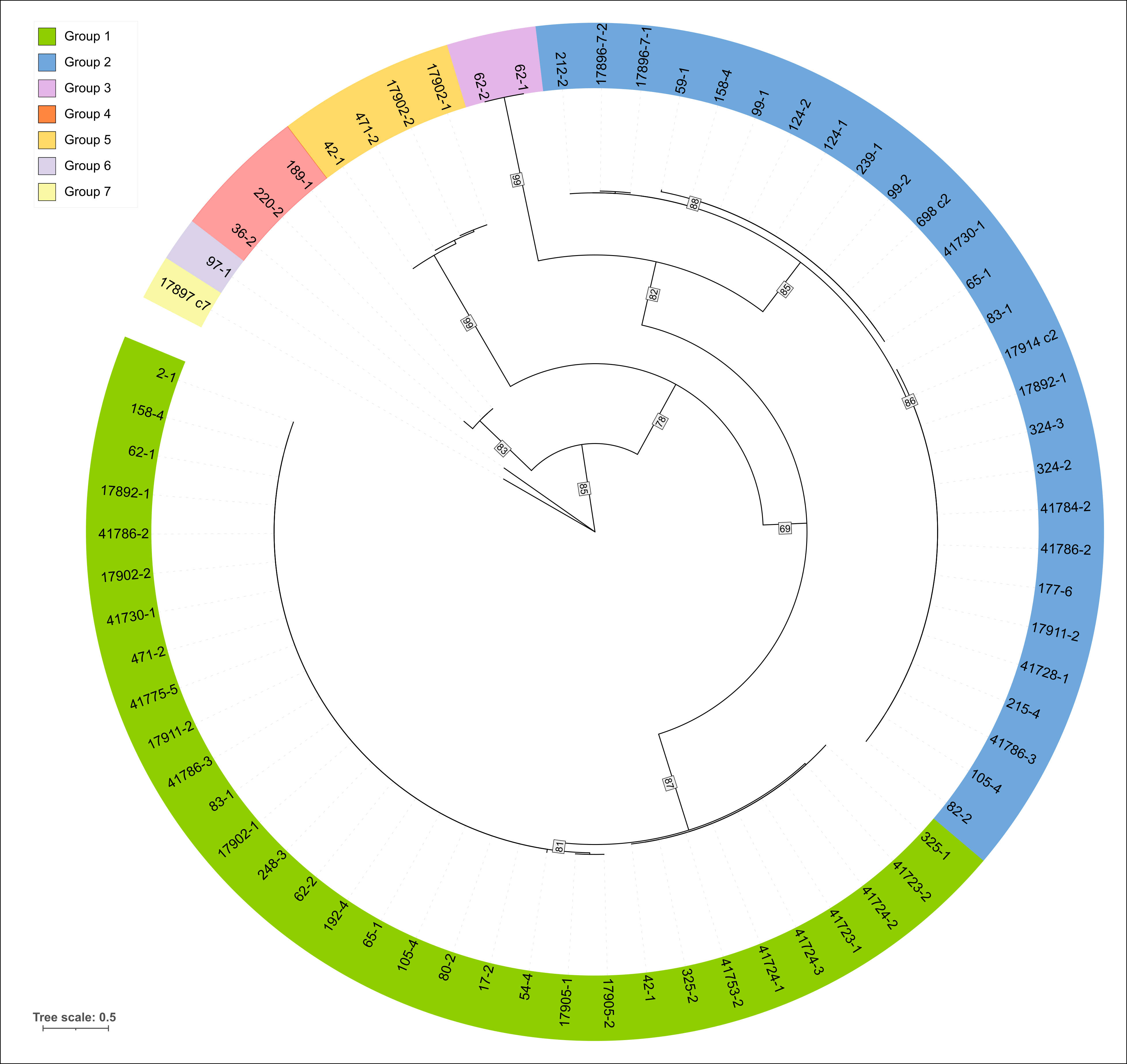

Supplement: Supplementary file 1 [file viruses-15-02215-s001.zip › Figure_S1.jpg]

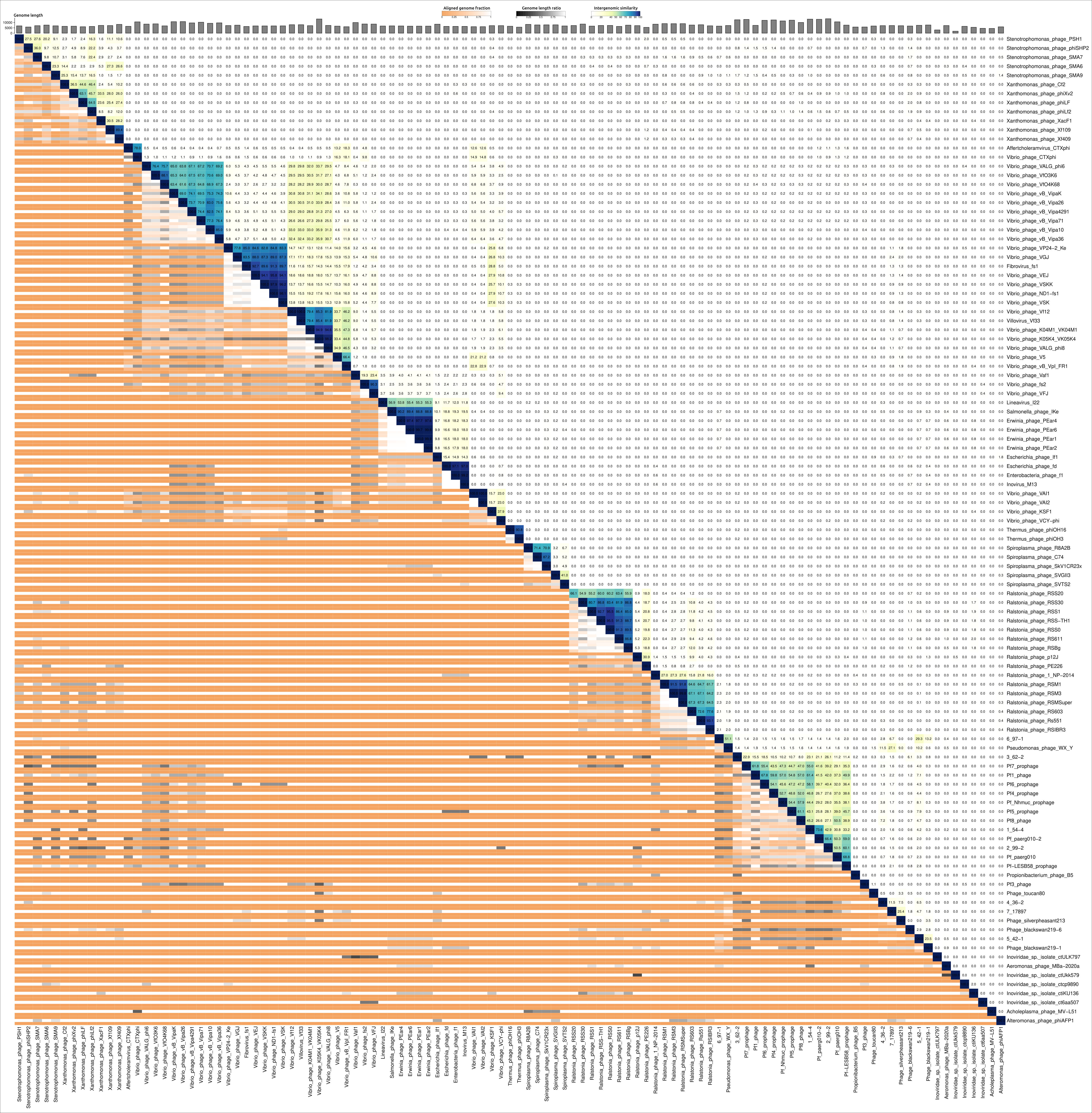

Supplement: Supplementary file 1 [file viruses-15-02215-s001.zip › Figure_S2.jpg]
